# Intracellular and non-neuronal targets of voltage-gated potassium channel complex antibodies

**DOI:** 10.1136/jnnp-2016-314758

**Published:** 2017-01-23

**Authors:** Bethan Lang, Mateusz Makuch, Teresa Moloney, Inga Dettmann, Swantje Mindorf, Christian Probst, Winfried Stoecker, Camilla Buckley, Charles R Newton, M Isabel Leite, Paul Maddison, Lars Komorowski, Jane Adcock, Angela Vincent, Patrick Waters, Sarosh R Irani

**Affiliations:** 1 Nuffield Department of Clinical Neurosciences, University of Oxford, Oxford, UK; 2 Institute for Experimental Immunology, Lubeck, Germany; 3 Department of Psychiatry, University of Oxford, Oxford, UK; 4 Department of Neurology, Queen's Medical Centre, Nottingham, UK

## Abstract

**Objectives:**

Autoantibodies against the extracellular domains of the voltage-gated potassium channel (VGKC) complex proteins, leucine-rich glioma-inactivated 1 (LGI1) and contactin-associated protein-2 (CASPR2), are found in patients with limbic encephalitis, faciobrachial dystonic seizures, Morvan's syndrome and neuromyotonia. However, in routine testing, VGKC complex antibodies without LGI1 or CASPR2 reactivities (double-negative) are more common than LGI1 or CASPR2 specificities. Therefore, the target(s) and clinical associations of double-negative antibodies need to be determined.

**Methods:**

Sera (n=1131) from several clinically defined cohorts were tested for IgG radioimmunoprecipitation of radioiodinated α-dendrotoxin (^125^I-αDTX)-labelled VGKC complexes from mammalian brain extracts. Positive samples were systematically tested for live hippocampal neuron reactivity, IgG precipitation of ^125^I-αDTX and ^125^I-αDTX-labelled Kv1 subunits, and by cell-based assays which expressed Kv1 subunits, LGI1 and CASPR2.

**Results:**

VGKC complex antibodies were found in 162 of 1131 (14%) sera. 90 of these (56%) had antibodies targeting the extracellular domains of LGI1 or CASPR2. Of the remaining 72 double-negative sera, 10 (14%) immunoprecipitated ^125^I-αDTX itself, and 27 (38%) bound to solubilised co-expressed Kv1.1/1.2/1.6 subunits and/or Kv1.2 subunits alone, at levels proportionate to VGKC complex antibody levels (r=0.57, p=0.0017). The sera with LGI1 and CASPR2 antibodies immunoprecipitated neither preparation. None of the 27 Kv1-precipitating samples bound live hippocampal neurons or Kv1 extracellular domains, but 16 (59%) bound to permeabilised Kv1-expressing human embryonic kidney 293T cells. These intracellular Kv1 antibodies mainly associated with non-immune disease aetiologies, poor longitudinal clinical–serological correlations and a limited immunotherapy response.

**Conclusions:**

Double-negative VGKC complex antibodies are often directed against cytosolic epitopes of Kv1 subunits and occasionally against non-mammalian αDTX. These antibodies should no longer be classified as neuronal-surface antibodies. They consequently lack pathogenic potential and do not in themselves support the use of immunotherapies.

## Introduction

Radioiodinated α-dendrotoxin (^125^I-αDTX) binds to neuronal voltage-gated potassium channels (VGKC) of the *Shaker*-family (Kv1.1, Kv1.2 and Kv1.6),^1^ and was used to label these channels in the radioimmunoassay which first identified VGKC complex autoantibodies in patients with neuromyotonia (NMT),^2^ and then in Morvan's syndrome (MoS),^3^ limbic encephalitis (LE)^4^ ^5^ and faciobrachial dystonic seizures (FBDS).^6^ ^7^ These autoantibodies were assumed to be directed against the Kv1 subunits themselves, but subsequent studies showed that the Kv1 subunits were part of a multiprotein neuronal complex that includes leucine-rich glioma inactivated 1 (LGI1), contactin-associated protein 2 (CASPR2) and contactin-2. Almost all of the antibodies from patients with LE, FBDS or MoS, and some with NMT, are directed against the extracellular domains of LGI1 or CASPR2, and the antibodies often co-immunoprecipitate the ^125^I-αDTX-labelled Kv1 subunits from brain extracts.^7–10^ Patients with LGI1 or CASPR2 antibodies often respond very well to immunotherapies, and their antibody levels broadly correlate with clinical status.^7^ ^11^ ^12^

Therefore, there has been an increasing interest in diagnosing these autoimmune neurological diseases,^13^ ^14^ leading to large numbers of requests for testing in patients who are unlikely to have well-defined autoimmune syndromes. This has generated an increase in the number of patient serum IgGs which precipitate the VGKC complex but lack LGI1 or CASPR2 reactivity (‘double-negative’ samples). These double-negatives can account for up to 80% of samples with positive VGKC complex antibodies in studies which most closely recapitulate clinical practice.^15^ ^16^ Moreover, the clinical syndromes in the double-negative patients are diverse and include patients with pain syndromes,^17^ status epilepticus,^18^ acute and chronic epilepsies,^19^ ^20^ inflammatory polyradiculopathies,^21^ children with a variety of neuroinflammatory diseases,^22^ systemic and central nervous system (CNS)-directed infections,^23^ a few patients with Creutzfeldt-Jakob disease,^24^ and up to 5% of elderly clinic controls.^4^ This clinical heterogeneity has questioned both the pathological relevance of the antibodies and the justification for immunotherapies in these patients.^13^ ^14^ ^16^ ^25^

Some studies have suggested that higher titres of the double-negative VGKC complex antibodies help to increase the likelihood of pathogenicity.^22^ ^26^ However, until now, the few available studies of double-negative patients have classified these patients by their clinical features and relied on arbitrary non-validated diagnostic criteria, and the subjective retrospective response to immunotherapies.^16^ ^25^ ^26^ Here, to definitively determine whether double-negative VGKC complex antibodies have pathogenic potential, we explored the epitopes they bound, their titres and clinical associations across a large variety of clinical syndromes.

## Methods

### Patients studied

To assess the frequencies of VGKC complex antibodies in a large number of varied patient phenotypes, and include syndromes reported to associate with double-negative samples, 1131 sera were tested from nine groups, including those with: (1) known LE, FBDS, MoS or NMT, LGI1 or CASPR2 antibodies and VGKC complex antibody levels >400 pM (n=84);^7^ ^8^ (2) a consecutive clinic cohort known to have VGKC complex antibodies without LGI1/CASPR2 reactivities (n=27; detailed in online supplementary table S1, which included patients with encephalopathies (n=10), NMT (n=2), stiff person syndrome (n=2), psychiatric conditions (n=6), isolated amnesia (n=2), Parkinson's disease dementia (n=1), Guillain-Barre syndrome (n=1) and neuropathic pain (n=3)); (3) adult-onset epilepsies (n=582); (4) infectious diseases (n=107: herpes simplex virus encephalitis (n=29), varicella zoster virus encephalitis (VZVE, n=20), measles encephalitis (n=30) and malaria (n=28, 12 with cerebral involvement)); (5) dysautonomia (n=95); (6) Lambert-Eaton myasthenic syndrome (n=45); (7) Hu-antibodies (n=78); (8) healthy smokers (n=38) and (9) healthy laboratory controls (n=75). Approval for antibody studies was from the Oxfordshire Regional Ethical Committee A (07/Q1604/28).

10.1136/jnnp-2016-314758.supp1 supplementary data

### Laboratory techniques

VGKC complex antibodies were detected by a radioimmunoassay which uses ^125^I-αDTX (Perkin Elmer, USA) to label VGKC complexes from 2% digitonin-solubilised rabbit whole brain membranes.^2^ ^4^ In order to closely mimic these conditions, but detect antibodies exclusively against the αDTX-sensitive Kv1 subunits, Kv1.1-tranfected, Kv1.2-tranfected and Kv1.6-transfected human embryonic kidney 293T (HEK) cells were used in place of brain tissue to prepare the extracts. In other respects, the radioimmunoassays were identical. To see if results were confounded by antibodies binding the ^125^I-αDTX itself, the tissue/cell extracts were replaced by solubilisation buffer. In each case, 5 μL of patient serum was incubated with 50 μL brain extract, HEK cell extract or buffer overnight and precipitated with 50 μL antihuman immunoglobulin G (IgG; Binding Site). The cut-off for positivity based on the mean plus three SDs of results from 20 healthy control sera was 100 pM for VGKC complex antibodies, 80 pM for Kv1 subunit antibodies and 137 pM for antibodies against ^125^I-αDTX alone.

Culture and staining procedures for live hippocampal neurons, and for live cell-based assays (CBAs) to detect antibodies against LGI1, CASPR2, contactin-2, Kv1.1, Kv1.2 and Kv1.6 were performed as described previously.^8^ To validate CBA results, flow cytometry was performed with live Kv1-transfected HEK cells incubated with patient serum (1:20), and bound-IgG detected using a phycoerythrin-conjugated antihuman IgG secondary antibody. Samples were analysed on a LSRII flow cytometer with FlowJo V.10.0.8 software. Fixed CBAs were performed (at Euroimmun AG, Lübeck, Germany) with Kv1-transfected HEK cells, after fixation with 1.8% formalin followed by pure acetone, using a 1:10 dilution of patient serum from coded vials, with unblinding after study completion.

Commercial antibodies against the extracellular domain of Kv1.1 (Neuromab, 75/105), and intracellular domains of Kv1.1 (Chemicon, AB9782), Kv1.2 (Millipore, AB5924 and Neuromab 75/008) and Kv1.6 (Chemicon, AB5184 and Neuromab 75/012) were used for immunoprecipitation and CBA studies. Commercial antibodies against the extracellular domains of Kv1.2 or Kv1.6 were not available. Statistics were performed using GraphPad Prism V.6, and individual tests are stated below.

## Results

### Antibodies against the VGKC complex

**Figure 1:**
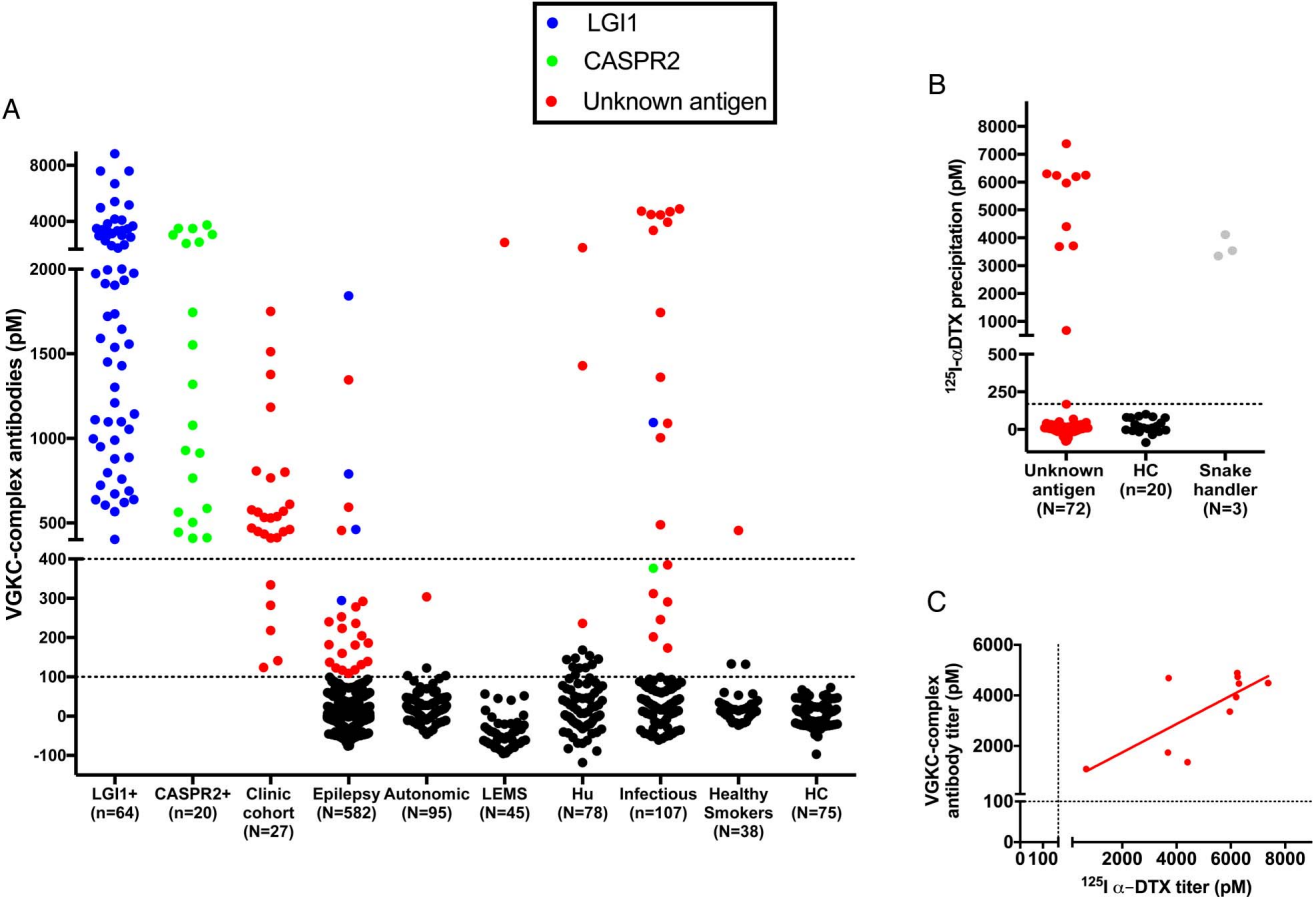
Detection of VGKC complex antibodies and antibodies to dendrotoxin. (A) VGKC complex antibodies were determined from 1131 samples, including those with known VGKC complex antibodies (both with (n=84) and without (n=27) LGI1 or CASPR2 reactivities), and unselected patients with adult-onset epilepsies, infectious diseases, autonomic syndromes, LEMS, Hu, healthy smokers and HC. Samples with LGI1 antibodies (n=69), CASPR2 antibodies (n=21) and all available samples with VGKC complex antibody levels above 100 pM and unknown antigenic targets (red; n=72) were carried forward to other assays. Dotted lines represent this cut-off and the 400 pM cut-off from a previous study;^4^ (B) 10 of the 72 samples with unknown antigens immunoprecipitated substantial quantities of of ^125^I-αDTX alone (dotted line at 137 pM represents the mean plus three standard deviations from 20 HCs). Three serum samples from a snake handler (grey dots) also had antibodies to ^125^I-αDTX; (C) ^125^I-αDTX antibody levels correlated with their corresponding VGKC complex antibody levels (r=0.54, p=0.015, Spearman's rank correlation). ^125^I-αDTX, radioiodinated α-dendrotoxin; CASPR2, contactin-associated protein-2; HC, healthy controls; Hu, Hu antibodies; LEMS, Lambert-Eaton myasthenic syndrome; LGI1, leucine-rich glioma-inactivated 1; VGKC, voltage-gated potassium channel.

Overall, across the varied cohorts, 162 of 1131 (14%) patients had VGKC complex antibodies, mainly from the groups with known positivity (figure 1A). Live CBAs showed that 90 of 162 (56%) patients had LGI1 or CASPR2 antibodies (4 with coexistent contactin-2 antibodies). Eighty-four of these 90 (90%) patients had LE, FBDS, MoS and NMT, and 80 of the 90 sera (87%), those with higher titres, also had IgG antibodies that bound the surface of hippocampal neurons. Of the remaining 72 (44%) double-negative VGKC complex antibody-positive samples, only one—from a patient with LE in the clinic cohort (see online supplementary table S1)—bound to live hippocampal neurons, suggesting a possible novel surface antigen. Among the cohorts (3–9) without known VGKC complex antibodies, the percentage of positives ranged between 0% and 4%, with the exception of the infectious group (19%) some of which had very high titres (figure 1A).

### Antibodies against ^125^I-αDTX

One possibility was that the double-negative sera bound to ^125^I-αDTX which is used to radiolabel the VGKC complex. Indeed, 10 of the 72 (14%) double-negative samples immunoprecipitated very high levels of the ^125^I-αDTX itself (figure 1B), correlating broadly with the corresponding VGKC complex antibody titres (figure 1C, r=0.54; p=0.02, Spearman's rank correlation). All these 10 were found in patients with infectious diseases (malaria (n=4), cerebral malaria (n=4), VZVE (n=1) and measles encephalitis (n=1)). Similarly, three sera from a snake handler, with vocational exposure to αDTX, immunoprecipitated ^125^I-αDTX (figure 1B). The remaining 62 double-negative samples without ^125^I-αDTX reactivity were carried forward to the next experiments.

### Antibodies against the αDTX binding VGKC subunits detected in solution

**Figure 2:**
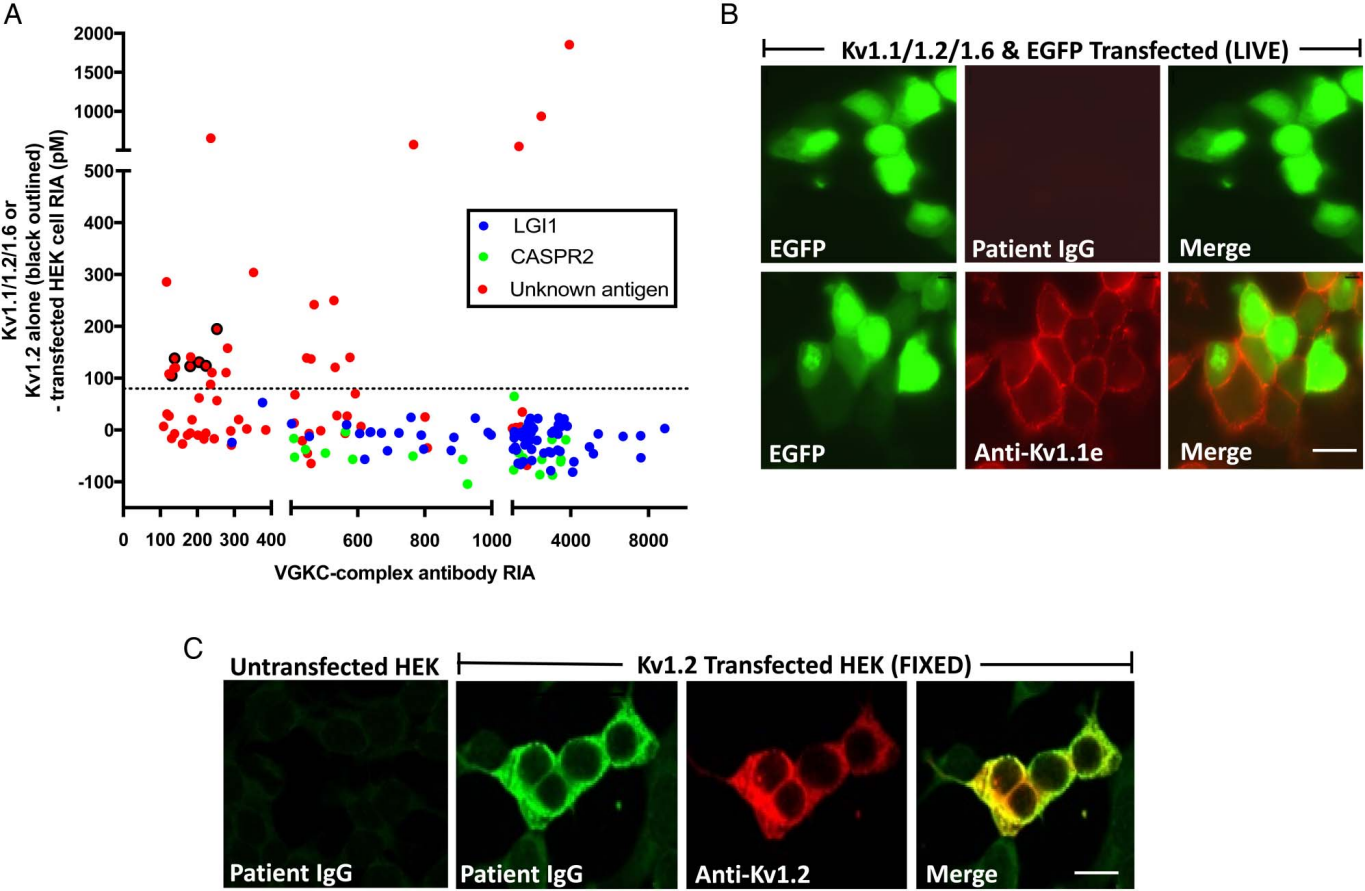
Kv1 antibodies target intracellular epitopes. (A) Twenty-seven of the remaining 62 patients with unknown VGKC complex antigenic targets precipitated either ^125^I-αDTX-labelled Kv1.1/Kv1.2/Kv1.6 co-transfected HEK cell extracts (red circles) or ^125^I-αDTX-labelled Kv1.2-transfected HEK cell extracts (red circles with black outline). No sera with LGI1 or CASPR2 antibodies showed positive results. HEK cells transfected with Kv1.1 alone or Kv1.6 alone did not bind ^125^I-αDTX in solution; (B) a commercial antibody to the extracellular domain of Kv1.1 (anti-Kv1.1e) labelled the cell surface of live HEK cells co-transfected with Kv1.1, Kv1.2 and Kv1.6 (and enhanced green fluorescent protein (EGFP)). No patient antibodies (n=175, including the 62 double-negative samples without αDTX reactivity) showed similar binding to these live cells or live cells expressing only one of these subunits; (C) binding to fixed Kv1-transfected HEK cells was seen using serum samples which precipitated Kv1s from solution. This co-localised with binding of commercial antibodies against the intracellular domain of Kv1.2 (anti-Kv1.2). Examples for Kv1.2 and Kv1.6 are shown in online supplementary figure S1C. Scale bar=10 microns. ^125^I-αDTX, radioiodinated α-dendrotoxin; CASPR2, contactin-associated protein-2; HEK, human embryonic kidney 293T; LGI1, leucine-rich glioma-inactivated 1; VGKC, voltage-gated potassium channel.

To test selectively for binding to the Kv1 subunits themselves, under conditions of the VGKC complex antibody assay, ^125^I-αDTX-labelled digitonin extracts of Kv1-transfected HEK cells were examined. Strikingly, none of the LGI1 or CASPR2 antibody-positive sera precipitated the Kv1 subunits (figure 2A). In contrast, 27 of 62 (44%) double-negative samples bound in solution to ^125^I-αDTX-labelled Kv1.1/1.2/1.6 heteromers only (n=6), ^125^I-αDTX-labelled Kv1.2 homomers only (n=6) or to both (n=15; figure 2A and see online supplementary figure S1A). Furthermore, their binding to Kv1s correlated well with their corresponding VGKC complex antibody levels (figure 2A, r=0.57, p=0.0017, Spearman's rank correlation). Ten of these 27 samples (37%) had VGKC complex antibody levels over 400 pM. No increase in binding was observed with additional co-transfection of postsynaptic density protein 95 (PSD95, see online supplementary figure S1B), a putative Kv1-clustering molecule.^27^

### Antibodies do not bind the extracellular domains of αDTX-sensitive VGKCs

The Kv1 reactivities available in solution could include intracellular or extracellular epitopes. To restrict detection to extracellular epitopes, live Kv1-transfected CBAs were tested. ^125^I-αDTX surface-binding studies on live Kv1-transfected HEK cells (see online supplementary figure S1C) and commercial antibodies to the extracellular domain of Kv1.1 (figure 2B) confirmed adequate surface expression of Kv1.1, Kv1.2 and Kv1.6. However, none of the double-negative sera bound to live HEK cells transfected with individual Kv1.1, Kv1.2 or Kv1.6, or all three Kv1 subunits (representative example in figure 2B). To ensure maximal sensitivity,^28^ these negative results were confirmed using flow cytometry on the live HEK cells co-transfected with Kv1.1, Kv1.2 and Kv1.6: there was no evidence of surface binding in the 16 sera from figure 2A with the highest Kv1 antibody radioimmunoassay values (see online supplementary figures S1D–F).

### Antibodies bind the intracellular domains of αDTX-sensitive VGKCs

These results prove the existence of double-negative VGKC complex antibodies which bind Kv1 subunits in solution, but not Kv1 extracellular epitopes. Therefore, HEK cells expressing Kv1.1, Kv1.2 and Kv1.6 were fixed and permeabilised so that antibodies against intracellular epitopes could be detected. Commercial antibodies raised against intracellular sequences of all the αDTX-sensitive Kv1 subunits bound specifically to the appropriate fixed HEK cells (example in figure 2C and see online supplementary figure S2A,B), and their binding was abrogated after absorption of the commercial antibody with the immunising cytosolic Kv1 subunit peptide (shown for Kv1.2, see online supplementary figure S2A). This confirmed accessibility of antibodies to intracellular Kv1-subunit epitopes.

**Figure 3:**
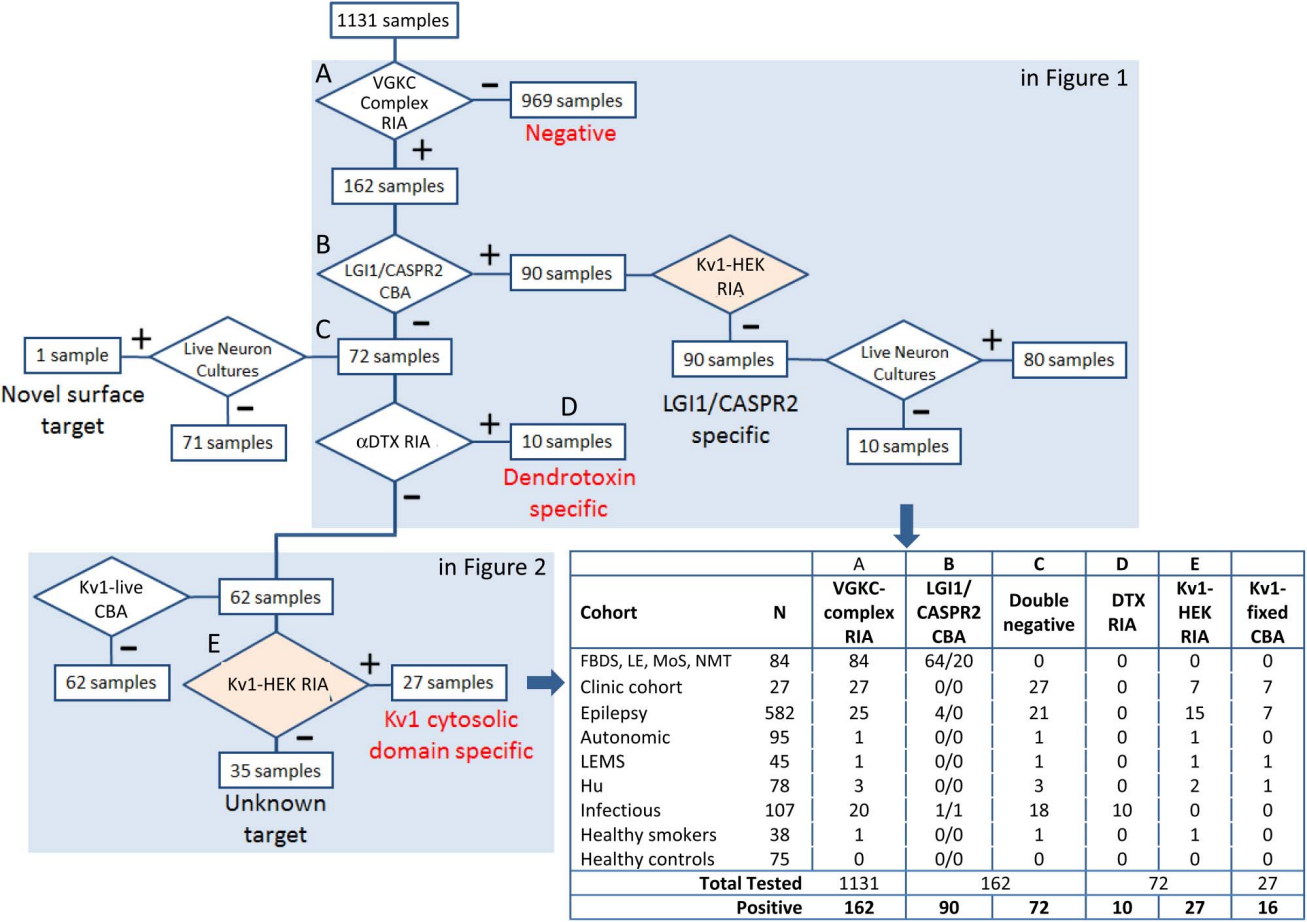
Summary of the sequential flow of assays through the study. As shown in figure 1A, 1131 samples were initially tested for VGKC complex antibodies by RIA (VGKC complex RIA, A) and subsequently using LGI1 and CASPR2 antibody live CBAs (B), live neuronal cultures (C) and precipitation of αDTX (αDTX RIA, D). As detailed in figure 2, double-negative samples were then tested for binding to the extracellular domains of live Kv1-tranfected HEK cells (Kv1-live CBA), for immunoprecipitations of ^125^I-αDTX-labelled Kv1-transfected HEK cells (Kv1-HEK RIA, E) and for binding to fixed permeabilised Kv1-transfected HEK cells (Kv1-fixed CBA). Cohorts are defined in more detail in the Methods section and online supplementary table S1. ^125^I-αDTX, radioiodinated α-dendrotoxin; CASPR2, contactin-associated protein-2; CBA, cell-based assay; HEK, human embryonic kidney 293T; Hu, Hu antibodies; LEMS, Lambert-Eaton myasthenic syndrome; LGI1, leucine-rich glioma-inactivated 1; RIA, radioimmunoassay; VGKC, voltage-gated potassium channel.

**Figure 4:**
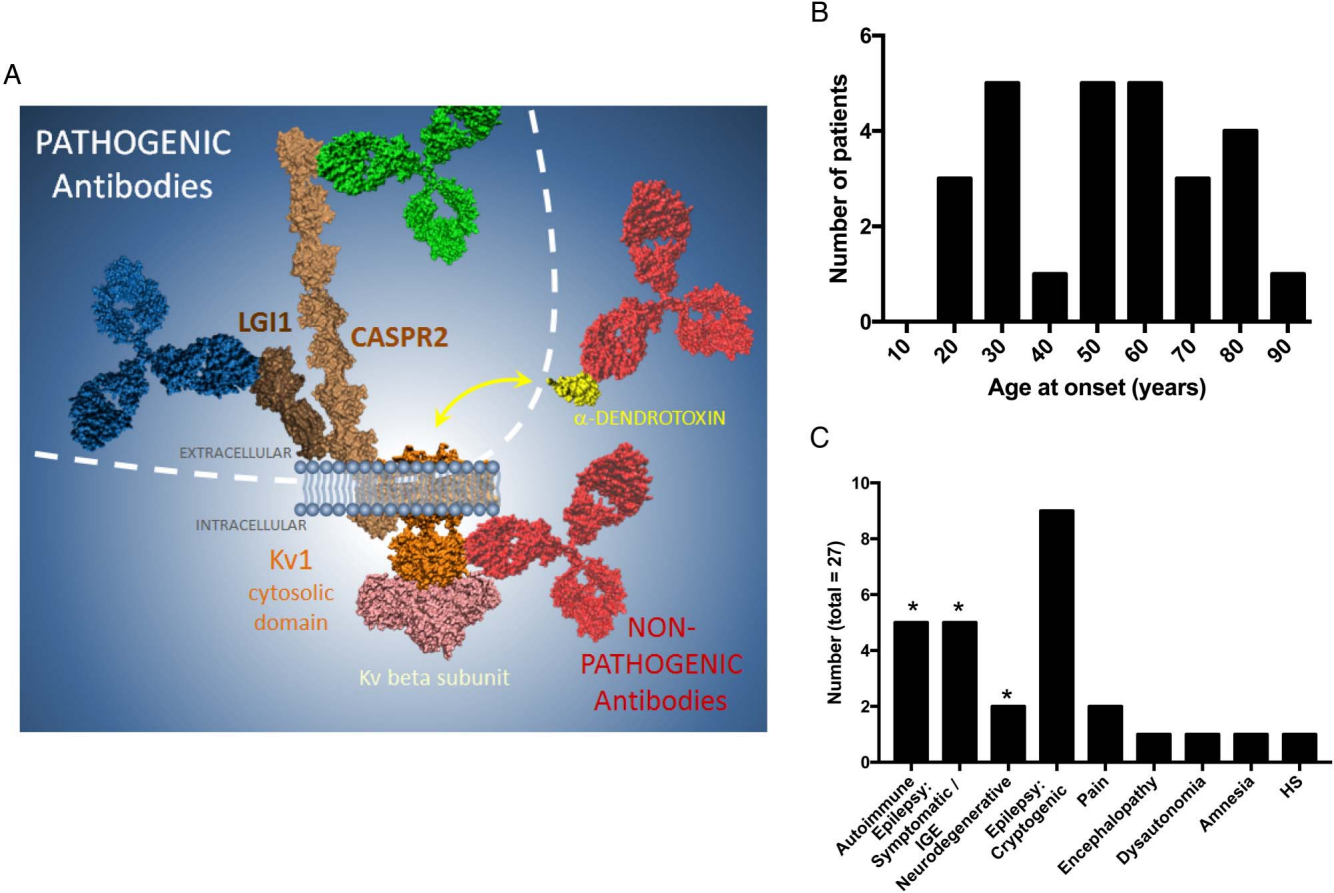
Molecular and clinical features associated with double-negative VGKC complex antibodies. (A) The illustration of study results demonstrates that the antibodies with pathogenic potential (blue and green) target the extracellular domains of LGI1 and CASPR2, respectively, whereas likely non-pathogenic antibodies (red) target the intracellular domain of Kv1 channels, especially Kv1.2, and the α-dendrotoxin molecule itself (yellow), which is not present in mammalian tissue. Other intracellular targets may include the Kv-β2 subunit (pink). (B) The patients with intracellular Kv1 antibodies had no clear peak age of onset, and (C) 12 showed varied, known diagnoses (*), and 15 had conditions of unknown aetiology, unlikely to be autoimmune. CASPR2, contactin-associated protein-2; HS, hippocampal sclerosis; LGI1, leucine-rich glioma-inactivated 1; VGKC, voltage-gated potassium channel.

Subsequently, 175 coded sera were tested for binding to the fixed permeabilised Kv1-expressing cells. These included first samples of all double-negative patients without αDTX reactivity (n=62), 6 with αDTX antibodies, 57 sequential samples from the 27 patients with Kv1 antibodies demonstrated in solution (from figure 2A), patients with known LGI1 and CASPR2 antibodies (n=20), and disease and healthy controls without VGKC complex antibodies (n=30). Binding was observed in 41 of 175 (23%) samples (examples in figure 2C and online supplementary figure S2A, B): all of these were from the group of 27 patients whose first sample immunoprecipitated Kv1.1/Kv1.2/Kv1.6 subunits (from figure 2A, Mann-Whitney test, p<0.0001). The first available sample from 16 of these 27 patients bound to Kv1.2 (n=9), Kv1.6 (n=3), Kv1.1 (n=1), Kv1.1, Kv1.2 and Kv1.6 (n=2), or both Kv1.2 and Kv1.6 (n=1). These patient IgGs showed consistent co-localisation with the Kv1 commercial antibodies (see online supplementary figure S2B). Overall, of the 27 samples which precipitated ^125^I-αDTX-labelled Kv1 subunits in solution, those which did not show fixed CBA positivity tended towards lower VGKC complex levels (Mann-Whitney test, p=0.001; see online supplementary figure S3A). The overall flow of samples and results by cohort is described in figure 3, and the extracellular and intracellular molecular reactivities of the VGKC complex antibodies are summarised in figure 4A.

### Correlations between Kv1 antibodies, clinical features and treatment responses

Serological and clinical details of the 27 patients with intracellular Kv1 antibodies are summarised in table 1. There were 16 men and 11 women, with ages ranging from 18 to 85 years and no peak age at onset (figure 4B). Only 5 (19%) had classical autoimmune syndromes (paraneoplastic, NMT or LE; figure 4C). The other patients had symptomatic or idiopathic generalised epilepsies (n=5), neurodegenerative diseases (n=2, Parkinson's disease dementia and Alzheimer's disease), and 14 presented with conditions of unknown aetiology including cryptogenic epilepsies (n=9), neuropathic pain (n=2), chronic encephalopathy (n=1), dysautonomia (n=1) or spontaneously resolving amnesia (n=1). In addition, one healthy smoker had intracellular Kv1 antibodies.

**Table 1:** Clinical–serological details of patients with antibodies against the intracellular aspects of Kv1.1, Kv1.2 and/or Kv1.6

	Serological results			Clinical features	Disease aetiology	Outcome and treatments	Summary of clinical–serological correlations
Age/Sex	VGKC complex antibody (pM)	Kv1-HEK RIA	Kv1-fixed CBA				
59/M	1346	+	1.2 only	Cryptogenic TLE with amnesia/depression	Unknown	No sustained response to three AEDs and CS	Poor. Persistent VGKC-c Abs despite markedly varied SZ frequencies. Online supplementary figure S3C
49/F	767	+	1.2 only	NMT with EMG confirmation	Autoimmune	Limited response to AEDs and CS	Poor. Highest VGKC-c Abs during clinical remission
55/M	533	+	1.2 only	NMT with EMG confirmation	Autoimmune	Symptoms over 8 years despite AEDs/AZA/CS	Good. Patient symptomatic with persistent VGKC-c Abs
77/M	470	+	1.2 only	Chronic myelopathy/encephalopathy	Unknown	Transient response to CS and PLEX	Poor. High VGKC-c Abs despite symptom fluctuations over 3 years
21/M	461	+	1.2 only	Cryptogenic probable frontal lobe epilepsy	Unknown	Ongoing SZs despite two AEDs	Poor. Online supplementary figure S3E
50/M	448	+	1.2 only	Isolated amnesia	Unknown	Spontaneous resolution over a few days	Poor. Abs reduced over 12 months. Symptoms resolved at 7 days
28/F	278	+	1.2 only	Cryptogenic TLE	Unknown	SZ freedom after first AED	Poor. Persistent VGKC-c Abs during SZ freedom
62/M	236	+	1.2 only	Cryptogenic TLE with amnesia	Unknown	SZs and amnesia at 5 years with 3 AEDs	Poor. VGKC-c Abs disappeared while SZs were ongoing
18/M	141	+	1.2 only	Limbic encephalitis	Autoimmune	Good response to CS and PLEX	Good. VGKC-c Abs reduced and clinical improvement at 12 months
25/F	529	+	1.6 only	Diffuse neuropathic pain and depression	Unknown	No response to opioids, CS or IVIG	Poor. High VGKC-c Abs despite marked fluctuations in symptoms
30/F	117	+	1.6 only	Idiopathic generalised epilepsy	Genetic	Good response to AEDs	NA
68/M	240	+	1.6 only	Alzheimer's disease; one SZ	Degenerative	Ongoing fall in memory despite CS/IVIG	Poor. Slight fall in VGKC-c Abs but marked reduction in memory over 4 years
34/M	577	+	1.2 and 1.6	Widespread neuropathic pain	Unknown	No response to CS, IVIG and PLEX	Poor. Constant pain despite highly varied VGKC-c Abs. Online supplementary figure S3D
71/F	2489	+	1.1, 1.2 and 1.6	NMT plus SCLC	Paraneoplastic	Palliative care only	NA.
67/F	2120	+	1.1, 1.2 and 1.6	LEMS plus Hu antibody neuropathy and SCLC	Paraneoplastic	Good response to CS	Poor. Persistently high VGKC-c Abs over 5 years despite clinical improvements
84/F	282	+	1.1 only	Parkinson's disease dementia	Degenerative	No response to CS	Poor. VGKC-c Abs reduced over 12 months despite clinical worsening
58/M	304	+	Negative	Dysautonomia	Unknown	NA	NA
37/M	253	+	Negative	TLE related to left HS	Structural	NA	NA
59/M	236	+	Negative	Healthy smoker	Healthy	Not relevant	Not relevant
48/F	223	+	Negative	TLE related to left HS	Structural	NA	NA
85/F	205	+	Negative	Cryptogenic focal motor SZs	Unknown	SZ free on 1 AED. Amnesia and anxiety benefited from CS/IVIG	Poor. VGKC-c Abs disappeared over 1 year; SZ freedom at 2 years. VGKC-c Abs returned at 4 years without SZs
33/F	182	+	Negative	Cryptogenic TLE	Unknown	SZ freedom after second AED	Moderate. VGKC-c Abs reduced over 6 months and SZ freedom at 1 year
76/M	181	+	Negative	Cryptogenic epilepsy	Unknown	SZ freedom with 1 AED	Poor. SZ freedom at 6 months; VGKC-c Abs sampled after 15 years
54/F	139	+	Negative	Epilepsy after childhood meningitis	Structural	NA	NA
22/M	137	+	Negative	Cryptogenic epilepsy	Unknown	SZ freedom after 1 AED	NA
54/M	131	+	Negative	Cryptogenic epilepsy	Unknown	SZ free at 1 year with 1 AED	Moderate. SZ free at 1 year; VGKC-c Abs absent at 3 months
77/M	123	+	Negative	TLE secondary to CVA	Structural	Ongoing SZs at 4 years	NA

Patients grouped by the VGKC-c antibody levels, precipitation from Kv1.1/Kv1.2/Kv1.6-cotransfected HEK cell and Kv1.2-transfected HEK cell radioimmunoassays (Kv1-HEK RIA; denoted as positive (+) or negative (−)) and the Kv1 subunit expressed fixed CBAs (Kv1-fixed CBA). All these patients had negative results in live CBAs for antibodies against Kv1s, LGI1, CASPR2, contactin-2 and for binding to live hippocampal neurons. Two patients had SCLC and tumours were not found in the remaining patients.

Ab, antibody; AEDs, antiepileptic drugs; AZA, azathioprine; CASPR2, contactin-associated protein-2; CS, corticosteroids; CBA, cell-based assay; CVA, cerebrovascular accident; EMG, electromyography; F, female; HEK, human embryonic kidney 293T; HS, hippocampal sclerosis; IVIG, intravenous immunoglobulins; LEMS, Lambert-Eaton myasthenic syndrome; LGI1, leucine-rich glioma-inactivated 1; M, male; NA, not available (only single serum sample obtained); PLEX, plasma exchange; SCLC, small cell lung carcinomas; SZ, seizure; TLE, temporal lobe epilepsy; VGKC, voltage-gated potassium channel; VGKC-c, VGKC complex.

Despite this serological and clinical heterogeneity, 6 of the 9 (67%) patients with cryptogenic epilepsies had Kv1.2-specific antibodies, both patients with neuropathic pain had Kv1.6 reactivities, and both patients with small cell lung carcinoma had antibodies directed against all three subunits (Kv1.1, Kv1.2 and Kv1.6).

Eleven of the 27 patients were administered immunotherapies and only 3 (27%) showed a sustained clinical benefit. In addition, only 4 of the 19 (21%) patients with serial serum samples demonstrated a relationship between intracellular antibody levels and clinical outcome (LE, NMT and 2 with cryptogenic epilepsies), while the remaining 15 demonstrated poor correlations. This contrasts with the majority of patients with LGI1 or CASPR2 antibodies (see online supplementary figure S3B–E). Interestingly, in all individual patients, the targeted Kv1 subunit(s) and their levels relative to VGKC complex antibodies remained constant over time, strongly suggesting that these two assays were measuring the same antibody populations (see online supplementary figures S3 C–E).

## Discussion

Autoantibodies directed against the extracellular domains of LGI1 and CASPR2 usually associate with distinctive immunotherapy-responsive syndromes. Indeed, clinical and accumulating paraclinical data strongly suggest that they are directly pathogenic.^8^ ^9^ ^11^ ^29–31^ In contrast, concerns have been raised about the clinical relevance of the double-negative VGKC complex antibodies that do not bind either of these proteins, particularly as they can be found in a proportion of patients with diseases which are unlikely to be of autoimmune aetiology.^13^ ^14^ ^16^ ^22^ ^25^ Studies which have interpreted the clinical relevance of such antibody results are limited by the inevitable difficulties in defining what constitutes an autoimmune disease.^16^ ^22^ ^25^ ^32^ Ultimately, the definition of autoimmune neurological diseases relies on the demonstration of a pathogenic immune factor. Therefore, the findings described here are important because they use a systematic biochemical approach to demonstrate conclusively that a proportion of VGKC complex antibodies binds to intracellular VGKC epitopes, or to the non-mammalian-expressed αDTX. Discovery of these important antigenic targets should influence ongoing clinical practice, and prompt re-evaluation of several reports describing the clinical associations of patients with double-negative VGKC complex antibodies.^15^ ^17–19^ ^26^ ^33^ In this study, double-negative antibodies were observed in several non-autoimmune conditions including highly variable central and peripheral nervous system syndromes with limited responses to immunotherapies, and poor correlations were noted between clinical data and serial antibody levels. Taken together, these double-negative autoantibodies often have targets which are inaccessible or non-existent in vivo, and they appear to be associated with limited clinical relevance.

Overall, LGI1 and CASPR2 antibody CBAs conferred greater clinical utility and better sensitivity and specificity than VGKC complex antibody testing in providing a diagnosis and rationale for immunotherapy. This should prompt clinicians to use LGI1 and CASPR2 antibodies over VGKC complex antibodies as first-line testing for pathogenic antibody-associated immunotherapy-responsive syndromes, and limit immunotherapy administration to patients with double-negative antibodies and atypical clinical syndromes.

A striking finding was the discovery of antibodies against αDTX itself both in 10 patients with CNS infections and in a snake handler. αDTX is a polypeptide toxin found in dendroaspis snake venoms and was essential for labelling the VGKC complex in the earliest studies.^2^ ^4^ ^5^ ^15^ ^34^ Some of these studies included control tests to avoid detecting αDTX antibodies,^15^ but commercial VGKC complex antibody assays do not provide this specific control. The αDTX antibodies were almost exclusively in sera with high titres of VGKC complex antibodies. In contrast, the intracellular epitopes identified on the Kv1 subunits themselves, particularly on Kv1.2, were found both at high and low VGKC complex antibody levels. Therefore, this study shows that the antigenic target—rather than the VGKC complex antibody level—aids prediction of potential pathogenicity. This is consistent with the observation that while VGKC complex antibody levels associated with LGI1 and CASPR2 reactivities are often high, they can be low or even undetectable.^8^ ^15^ ^25^ ^35^ ^36^

Furthermore, this is the first clear demonstration of double-negative VGKC complex antibodies which target intracellular epitopes. This positive demonstration is important as we and others have previously hypothesised their existence, mainly inferring from the absence of live neuronal binding.^13^ ^14^ ^22^ ^25^ However, since a few disease-relevant LGI1 and CASPR2 antibody-positive samples had no detectable live hippocampal neuron binding, the absence of live neuronal binding should not necessarily imply the presence of intracellular reactivities. Indeed, it is most likely that not all surface neuronal proteins are expressed in a hippocampal cell culture system.

It is curious that the double-negative VGKC complex epitopes appear especially immunogenic: they are found after both human exposure to porcine brain aerosols and murine nasal immunisations with brain extracts,^21^ suggesting that these antibodies are easily induced. Collectively, alongside their presence in a variety of epilepsies, neurodegenerative diseases and idiopathic syndromes, these observations imply that double-negative antibodies arise secondary to diverse pathologies, where the primary pathological insult may not be immune. Indeed, perhaps non-immune causes of neuronal destruction are sufficient to release immunogenic Kv1 antigens and provoke autoantibody production.^16^ ^22^ ^26^ Interestingly, the immunotherapy response observed in 27% of patients is very similar to the placebo response rates observed in several other neurological diseases,^37^ and much lower than those reported in LGI1 or CASPR2 antibody-associated syndromes.^7–9^ ^11^ ^15^ ^29^ ^35^ However, conclusions regarding treatment outcomes are only definitive after blinded interventional studies. Also, the demographics of the patients with Kv1 antibodies did not show any age or sex bias, quite different from the occurrence of LGI1 antibody LE in later years or the male predominance of CASPR2 antibodies.^8^ ^9^ ^11^ ^29^ Alongside the 5% rate of double-negative VGKC complex antibodies in healthy individuals,^4^ these observations suggest that double-negative reactivities have very limited clinical significance.

On the other hand, we noted relationships between specific Kv1 subunit reactivities and clinical syndromes, particularly Kv1.2 antibodies in cryptogenic epilepsies, Kv1.6 antibodies in the two patients with neuropathic pain, and Kv1.1, Kv1.2 and Kv1.6 antibodies in paraneoplastic syndromes. These preliminary findings suggest that specific patterns of Kv1 reactivities may be generated in response to certain pathologies. Indeed, a few patients with classical VGKC complex antibody-associated syndromes, such as NMT and LE, did show good antibody-clinical correlations, and in these patients the Kv1 antibodies may be markers of a coexistent pathogenic antibody.^38^ ^39^ However, while many of the clinic cohort had an underlying immune basis to their neurological syndrome, ascertainment of patients attending a specialist autoimmune clinic may well carry this intrinsic bias.

In summary, our results show that many double-negative VGKC complex antibodies lack pathogenic potential, and that direct examination of LGI1 and CASPR2 antibodies is more informative than first-line VGKC complex antibody testing.^13^ ^14^ ^25^ ^35^ ^36^ Also, direct LGI1 and CASPR2 antibody CBAs appear more sensitive than VGKC complex antibody or live neuronal antibody determination.^25^ ^35^ ^36^ Therefore, direct LGI1 and CASPR2 antibody assays are the preferred tests for clinical purposes. This approach should limit the use of unnecessary immunotherapies in double-negative patients.

Until all centres update their practices, treatment of double-negative cases should be limited to those with a high pretest probability of an underlying autoimmune condition. However, for research purposes, it may be useful for further studies to identify the remaining, likely intracellular, double-negative VGKC complex antigens such as the Kv-β2 subunit and PSD95, and explore whether they are useful biomarkers of traditionally non-immune or immune neurological syndromes.
