# Supplementary material for: Intracellular and non-neuronal targets of voltage-gated potassium channel complex antibodies
Source: J Neurol Neurosurg Psychiatry. 2017 Jan 23;88(4):353–61. doi: 10.1136/jnnp-2016-314758 (PMC5644714; doi:10.1136/jnnp-2016-314758)
Supplement: supplementary data [file jnnp-2016-314758supp001.pdf]

Sex, age	VGKC-complex antibodies (pM)	Syndrome classification	Clinical and Investigation features	Treatments and responses
M, 18	141	Encephalopathy	Amnesia and seizures with hippocampal T2-high signal	Partial improvement after corticosteroids, IVIG and PLEX
M, 61	450	Encephalopathy	Ataxia, depression and confusion	Partial response to PLEX
F, 61	808	Encephalopathy	Amnesia and seizures with hippocampal T2-high signal	Good response to corticosteroids and PLEX. Antibodies to surface of live hippocampal neurons.
M, 17	461	Encephalopathy	Amnesia, confusion, hypersomnolence. Recurrent bouts.	No immunotherapies, unchanged
F, 24	1378	Encephalopathy	Coma, amnesia and confusion with CSF lymphocytic pleocytosis. Rapid spontaneous improvement.	No immunotherapy. Complete recovery
M, 70	538	Encephalopathy	Confusion, tremor and amnesia. Very high doses of sodium valproate.	Improvement after corticosteroids and PLEX
M, 29	411	Encephalopathy	Thyroid eye disease	No immunotherapies, mild improvement
M, 70	435	Encephalopathy	Amnesia, confusion, hallucinations, hypersomnolence	No immunotherapies, unchanged
M, 40	470	Encephalopathy	Amnesia, confusion, seizures plus brainstem involvement	Partial recovery without immunotherapies
M, 77	470	Encephalopathy	Confusion, seizures, amnesia plus myelopathy	Partial response to corticosteroids and PLEX
F, 49	767	Neuromyotonia	EMG proven neuromyotonia with mild relapse	Limited response to AEDs and corticosteroids
M, 61	533	Neuromyotonia	EMG proven neuromyotonia	Limited response to AEDs, corticosteroids and azathioprine

F, 54	801	Stiff person syndrome	Auditory and tactile startle. Hypothroidism.	Improvement with corticosteroids and PLEX
F, 27	646	Stiff person syndrome	Auditory startle and rigidity	Good response to IVIG
F, 70	218	Psychosis	Delusions, thought disorder and decline in function	No change with corticosteroids
F, 38	334	Psychosis	Delusions, thought disorder and hallucinations	No immunotherapy. Mild improvement
F, 62	1513	Psychosis	Delusions and thought disorder	No change with corticosteroids
M, 25	1184	Depression	Headaches and attentional deficits	No immunotherapies, unchanged
M, 37	413	Depression	Low mood	No change with corticosteroids
F, 27	124	Depression	Also, functional movement disorder	No immunotherapy. Mild improvement
M, 50	610	Amnesia only	Isolated amnesia after fever	Full recovery without immunotherapies
M, 50	448	Amnesia only	Spontaneously resolving amnesia over few weeks	No immunotherapy. Complete improvement
F, 85	282	Parkinson's disease dementia	Headaches and depression with later appearance of tremor	No immunotherapy. Mild progression
M, 83	4044	Guillian-Barre syndrome.	Demyelinating motor neuropathy.	Marked improvement with IVIG
F, 39	529	Neuropathic pain	Burning pains after idiopathic meningitis	No benefit from corticosteroids or IVIG
M, 21	577	Neuropathic pain	Burning, lancinating pains and depression	Mild improvement with corticosteroids, PLEX and cyclophosphamide
F, 26	1342	Neuropathic Pain	Burning multidermatomal pain; two seizures; raised CSF protein	No response to corticosteroids or IVIG

Supplementary Table 1. Clinical and paraclinical features of the clinic cohort: 27 patients with VGKC-complex antibodies lacking LGI1 and CASPR2 reactivities. None had tumors. AEDs = antiepileptic drugs; IVIG = intravenous immunoglobulins; PLEX = plasma exchange.

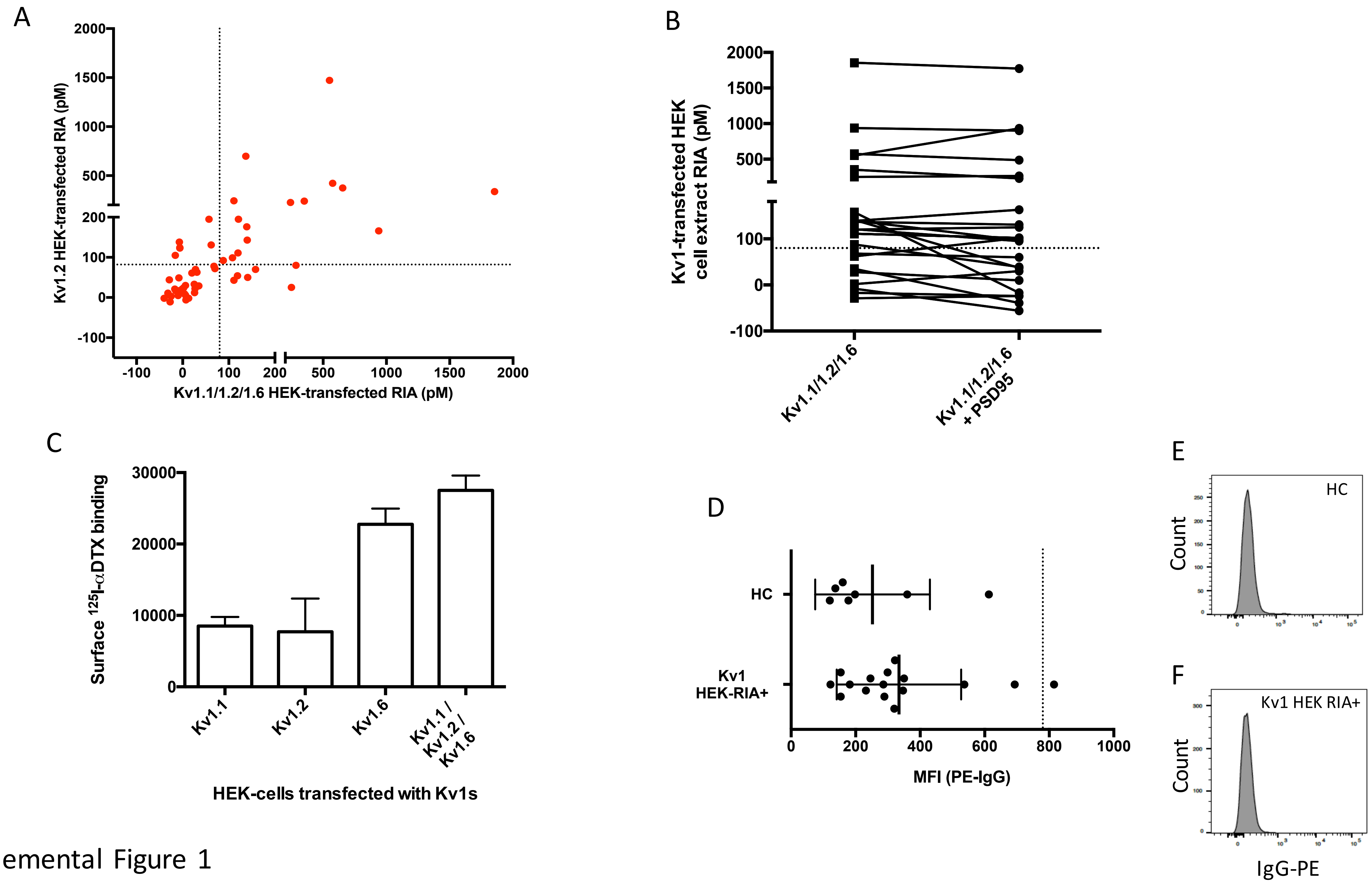

Supplemental Figure 1

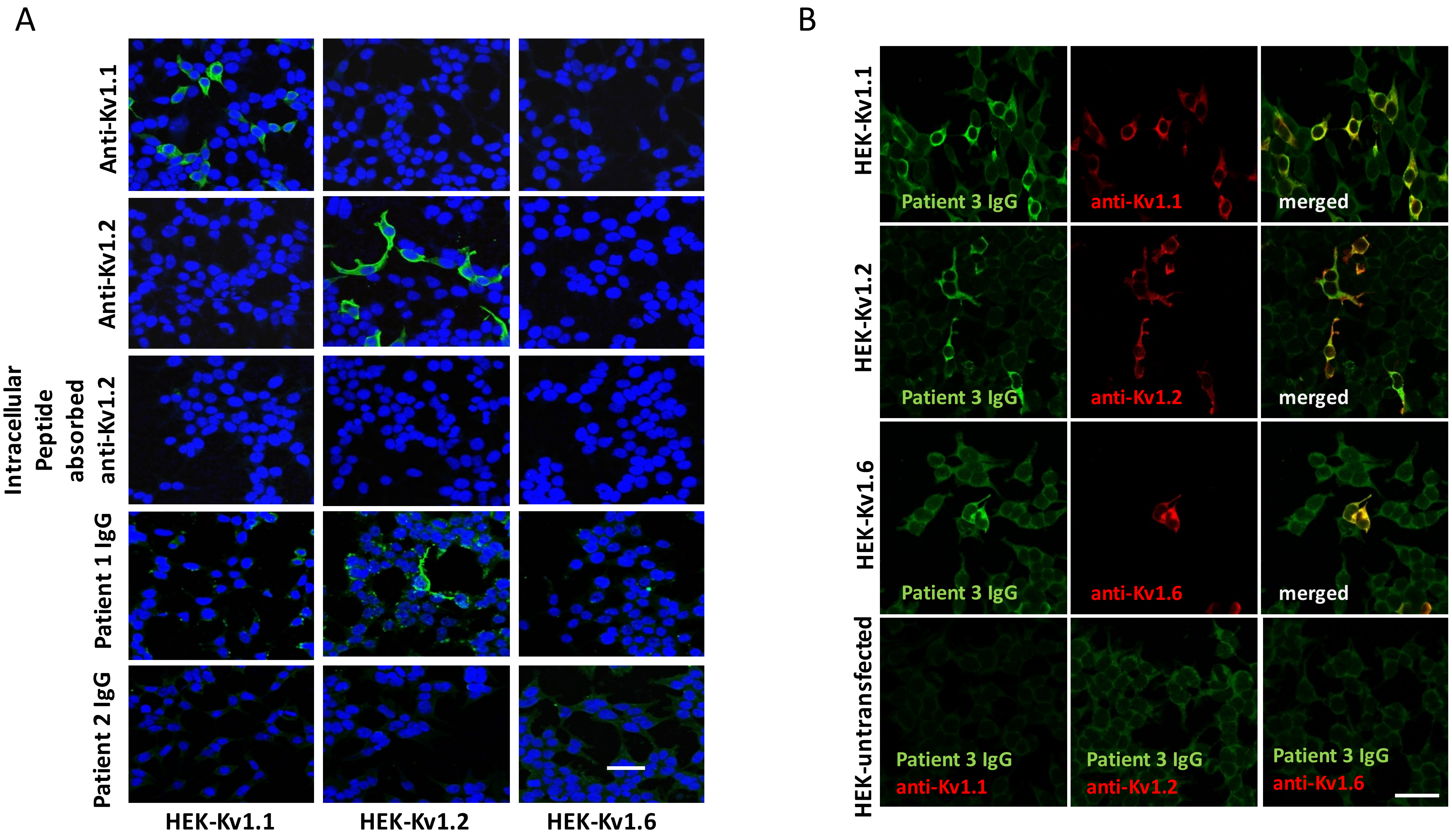

Supplemental Figure 2

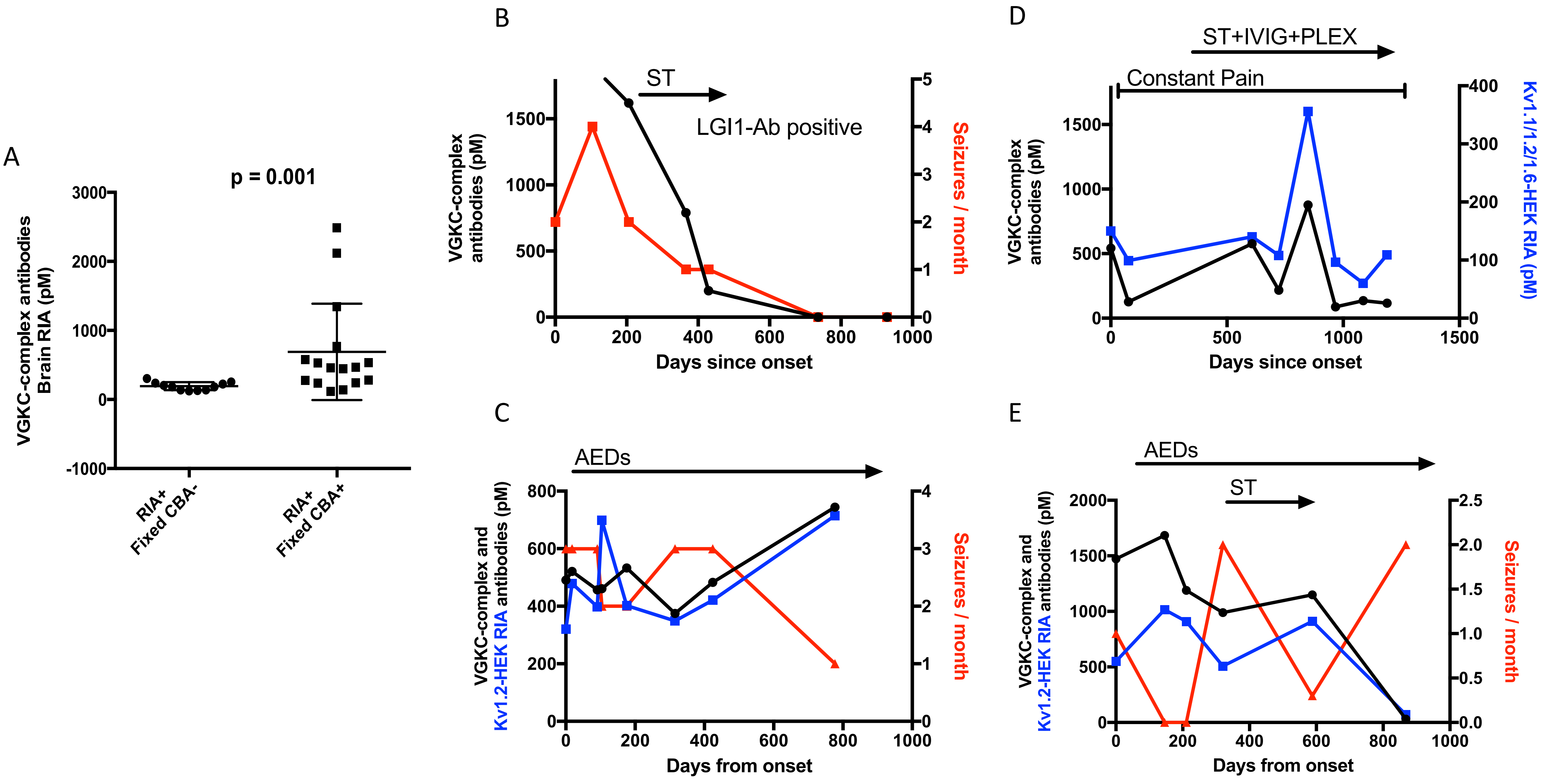

Supplemental Figure 3

Supplementary Figure 1. VGKC-complex double-negative antibody epitopes (A) Relative precipitations from ^{125}I-αDTX-labelled Kv1.1/1.2/1.6-co-transfected HEK-cell extracts and ^{125}I-αDTX-labelled Kv1.2-transfected HEK cell extracts showed 15 samples bound both preparations, and an additional 12 bound exclusively one or the other. Mean plus three standard deviations from healthy controls (n=20) generated a cut-off of 80 pM (dotted line); (B) No increase in binding was observed with additional co-transfection of postsynaptic density protein 95 (PSD95); (C) High levels of surface Kv1 expression (determined by ^{125}I-αDTX-binding) were observed for Kv1.1-, Kv1.2-, Kv1.6- and Kv1.1/1.2/1.6-co-transfected cells. (D) Using flow cytometry, the 16 samples with the greatest precipitation from ^{125}I-αDTX-labelled Kv1.1/1.2/1.6-co-transfected HEK cell extracts (Kv1 HEK RIA+) showed median fluorescent indices (MFI) of IgG binding which were equivalent to those of healthy controls (HC) by live Kv1.1/1.2/1.6-co-transfected HEK cell flow cytometry. The dotted line represents mean plus three standard deviations from HCs. Representative flow-cytometry histograms are shown for a HC (E), and a patient (F) with known Kv1-subunit antibodies in solution (Kv1 HEK RIA+). PE = Phycoerythrin.

Supplementary Figure 2. Intracellular Kv1 epitopes. (A) Commercial antibodies against Kv1.1 (anti-Kv1.1) and Kv1.2 (anti-Kv1.2) specifically bind HEK cells transfected with Kv1.1 (HEK-Kv1.1) and Kv1.2 (HEK-Kv1.2) respectively, without cross-reactivity (upper two rows). The binding was lost after the antibodies were absorbed against the immunizing peptide which represented an intracellular sequence of the corresponding Kv1-subunit peptide (data shown for Kv1.2: intracellular peptide absorbed anti-Kv1.2, middle row). Representative examples shown from a patient IgG which only bound the permeabilized Kv1.2-transfected HEK cells (fourth row, Patient 1), and patient IgG which showed no Kv1-subunit reactivity (fifth row, Patient 2). (B) Co-localization of commercial antibody (red, anti-Kv1.1, anti-Kv1.2 and anti-Kv1.6) and patient 3 IgG binding (green) to fixed HEK cells

transfected with Kv1.1 (HEK-Kv1.1), Kv1.2 (HEK-Kv1.2) or Kv1.6 (HEK-Kv1.6). No patient or commercial antibodies bound untransfected HEK cells. Merged images shown in right hand column. Scale bar = 50 microns.

Supplementary Figure 3. (A) Kv1-radioimmunoassy – Kv1-fixed CBA comparison. From all samples which were positive in the Kv1-subunit HEK-cell radioimmunoassays (RIA), the VGKC-complex antibody levels were higher in those with positive results by fixed CBA (CBA+; Mann Whitney test, p=0.001). Longitudinal seizure frequencies (red), VGKC-complex antibody levels (black) and Kv1.1/1.2/1.6 co-transfected or Kv1.2-transfected HEK-cell radioimmunoassay (RIA) results (blue) are shown (B) for a patient with epilepsy and LGI1-antibodies from Figure 1A, and three patients with intracellular Kv1-antibodies (C-E; cross-referenced with additional clinical details in Table 1). AEDs = antiepileptic drugs; IVIG = intravenous immunoglobulins; PLEX = plasma exchange; ST = corticosteroids.
